# Necroptosis in Immuno-Oncology and Cancer Immunotherapy

**DOI:** 10.3390/cells9081823

**Published:** 2020-08-01

**Authors:** Jenny Sprooten, Pieter De Wijngaert, Isaure Vanmeerbeek, Shaun Martin, Peter Vangheluwe, Susan Schlenner, Dmitri V. Krysko, Jan B. Parys, Geert Bultynck, Peter Vandenabeele, Abhishek D. Garg

**Affiliations:** 1 Department of Cellular and Molecular Medicine, Laboratory of Cell Stress & Immunity (CSI), KU Leuven, 3000 Leuven, Belgium; jenny.sprooten@kuleuven.be (J.S.); pieter.dewijngaert@student.kuleuven.be (P.D.W.); isaure.vanmeerbeek@kuleuven.be (I.V.); 2 Department of Cellular and Molecular Medicine, Laboratory of Cellular Transport Systems, KU Leuven, 3000 Leuven, Belgium; shaun.martin@kuleuven.be (S.M.); peter.vangheluwe@kuleuven.be (P.V.); 3 Department of Microbiology, Immunology and Transplantation, KU Leuven, 3000 Leuven, Belgium; susan.schlenner@kuleuven.be; 4 Department of Human Structure and Repair, Cell Death Investigation and Therapy Laboratory, Ghent University, 9000 Ghent, Belgium; Dmitri.Krysko@ugent.be; 5 Department of Pathophysiology, Sechenov First Moscow State Medical University (Sechenov University), Moscow 119146, Russia; 6 Department of Cellular and Molecular Medicine and Leuven Kanker Instituut (LKI), Laboratory of Molecular and Cellular Signaling, KU Leuven, 3000 Leuven, Belgium; jan.parys@kuleuven.be (J.B.P.); geert.bultynck@kuleuven.be (G.B.); 7 Department of Biomedical Molecular Biology, Ghent University, 9000 Ghent, Belgium; Peter.Vandenabeele@irc.vib-Ugent.be; 8 VIB Center for Inflammation Research, 9052 Ghent, Belgium; 9 Methusalem Program, Ghent University, 9000 Ghent, Belgium

**Keywords:** cytokines, interferons, danger signals, damage-associated molecular patterns (DAMPs), dendritic cells, macrophages, T cells, prognostic/predictive biomarkers, patients, immunogenic cell death

## Abstract

Immune-checkpoint blockers (ICBs) have revolutionized oncology and firmly established the subfield of immuno-oncology. Despite this renaissance, a subset of cancer patients remain unresponsive to ICBs due to widespread immuno-resistance. To “break” cancer cell-driven immuno-resistance, researchers have long floated the idea of therapeutically facilitating the immunogenicity of cancer cells by disrupting tumor-associated immuno-tolerance via conventional anticancer therapies. It is well appreciated that anticancer therapies causing immunogenic or inflammatory cell death are best positioned to productively activate anticancer immunity. A large proportion of studies have emphasized the importance of immunogenic apoptosis (i.e., immunogenic cell death or ICD); yet, it has also emerged that necroptosis, a programmed necrotic cell death pathway, can also be immunogenic. Emergence of a proficient immune profile for necroptosis has important implications for cancer because resistance to apoptosis is one of the major hallmarks of tumors. Putative immunogenic or inflammatory characteristics driven by necroptosis can be of great impact in immuno-oncology. However, as is typical for a highly complex and multi-factorial disease like cancer, a clear cause versus consensus relationship on the immunobiology of necroptosis in cancer cells has been tough to establish. In this review, we discuss the various aspects of necroptosis immunobiology with specific focus on immuno-oncology and cancer immunotherapy.

## 1. Introduction

Over the last decade it has become clear that, as compared to conventional anticancer therapies (such as radiotherapy, chemotherapy or targeted therapy), immune-based therapeutics have the highest probability of durably prolonging the survival of (at least a subset of) cancer patients with relatively good quality of life [1,2]. Immunotherapies, especially those antagonizing immune-checkpoints such as programmed cell death protein 1 (PD1) or cytotoxic T lymphocytes associated protein 4 (CTLA4) (both part of the family of immune-regulatory molecules crucial for general suppression of anti-tumor immunity) have been approved to treat several solid malignancies, such as melanoma, lung cancer, head and neck cancer, renal cell cancer and bladder cancer (among others) [3,4,5]. For the first time in decades, immune-checkpoint blockers (ICBs)-based immunotherapies have allowed oncologists to anticipate tumor curative strategies [6,7,8]. This transformation has been so drastic that ICBs have, in a very short period, replaced various standard-of-care conventional therapies to take the position of first line (neo)adjuvant therapies for melanoma, lung cancer and renal cell cancer [9,10].

Despite these advances, not all cancer patients durably respond to ICBs [11,12,13]. Resistance to ICBs is visible not only on the level of cancer types (e.g., ovarian cancer, glioblastoma, prostate cancer, pancreatic cancer, sarcomas), but also in subgroups of patients of cancer types typically responsive to immunotherapy (e.g., melanoma, lung cancer and renal cell cancer) [14,15]. Such resistance is often orchestrated through well-established immuno-evasive mechanisms propagated by cancer cells: (I) exhibition of low immunogenic potential (by reducing expression of cancer antigens or molecules involved in antigen presentation machinery) [16,17,18]; (II) provoking dysregulation of the lymphoid compartment through immunosuppressive cytokines [19,20], T lymphocytes-excluding factors [21] or even ‘alternative’ immune-checkpoints (i.e., immune-checkpoints other than PD1 or CTLA4) [22,23], thus enabling deletion of T cells through prolonged exertion of immunological and metabolic exhaustion [24,25,26]; (III) disrupting long-term differentiation as well as perseverance of memory T lymphocytes [27,28]; and, (IV) upregulation of regulated cell death (RCD)-resistance mechanisms that can also neutralize T cells-mediated cancer cell lytic activity [22,29].

Rescuing the effector potential of anticancer lymphocytes via T cells targeting (e.g., agonists of T cell activating molecules) or reconstituting (e.g., adoptive T cell transfer) therapies are being intensively explored to overcome these resistance mechanisms [30,31,32]. A complementary approach has been to promote the immunogenicity of cancers cells by disrupting tumor-associated immune-tolerance through conventional anticancer therapies [33,34,35,36,37]. It has been proposed that first-line application of conventional anticancer therapies (at highly calculated doses) may “reset” the tumor microenvironment through immune response-facilitating cancer cell death, thereby making it more susceptible to ICBs-driven effector T cell infiltrates [38,39,40,41]. This was exemplified by the recently published TONIC (Trial of Nivolumab after induction treatment in triple-negative breast cancer) clinical trial, wherein breast tumors pre-treated with cisplatin or doxorubicin responded favorably to second-line intervention with PD1-targeting ICBs [42]. Therefore, anticancer therapies that cause an immunogenic or even inflammatory demise of cancer cells are best positioned to productively “pulse” professional antigen-presenting cells (APCs) like dendritic cells (DCs) with cancer antigens [43,44,45,46]. This can further enable DCs-driven priming of T cells for these antigens, thereby creating antigen-specific effector T cells [47,48,49,50,51]. Despite the excitement about integrating conventional anticancer therapies that induce immunogenic or inflammatory sub-forms of RCD into immuno-oncology paradigms, there is a lack of consensus on which RCD sub-form should be prioritized in combinatorial regimens.

**Figure 1:**
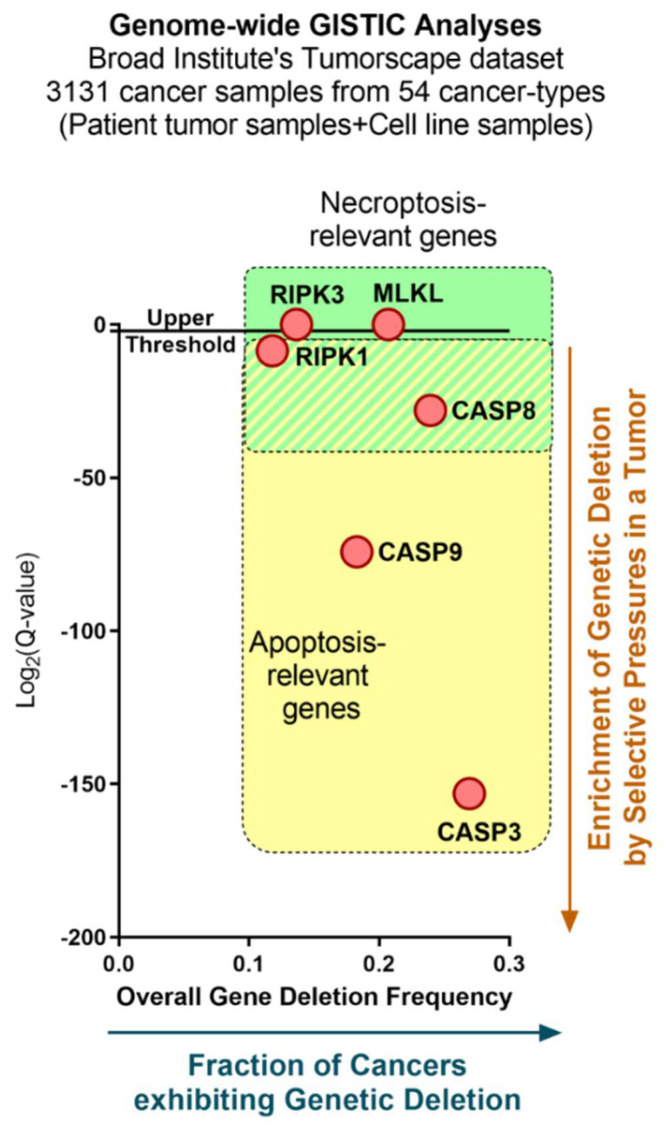
A genetic analysis of tumor-specific genetic “selection pressures” on apoptosis- and necroptosis-relevant genes. A Genomic Identification of Significant Targets in Cancer (GISTIC) analysis of DNA deletion based on the analysis of 3131 cancer samples from 54 cancer types using the Tumorscape (www.broadinstitute.org/tumourscape) database (accessed in May 2018). GISTIC scores (*X*-axis) and false-discovery rate or FDR (*Y*-axis; Q-values with 0.25 as cut-off for significance) for each alteration are plotted. GISTIC is an algorithm that strives to characterize putative cancer-driving somatic copy-number alterations (SCNAs) by analyzing the frequency as well as amplitude of the observed genetic events (e.g., deletions) [68]. Accordingly, GISTIC score (*X*-axis) provides a prediction of genetic deletion events under both loci-specific selection pressure as well as background genetic (random) deletion rates (which naturally tend to be very high in cancer). However, the Q-values (*Y*-axis) further allow the differentiation between the above two events, such that a significantly low Q-value signifies loci-specific selection pressures whereas a high Q-value signifies random genetic deletion events at the same rate as background genetic aberrations in cancer. For further details on this analysis’s methodology, we refer the reader to the publication by Mermel et al. [68]. Of note, whereas *RIPK1* is indeed a necrosome-relevant gene, its functions are not exclusive to necroptosis since it can also play differential role in apoptosis or survival.

Many studies exploring immunogenic or inflammatory sub-forms of RCD in cancer have emphasized the importance of apoptosis induced by various anticancer therapies [52,53]. Furthermore, apoptosis is a form of RCD that is associated with an orderly intracellular degradation of the dying cell that enables efficient immunological clearance and disposal [52]. Herein, specific chemotherapies (e.g., anthracyclines, oxaliplatin), targeted therapies (e.g., bortezomib), physical therapies (e.g., photodynamic therapy, high hydrostatic pressure) and oncolytic viruses have been reported to induce an immunogenic form of apoptosis or immunogenic cell death (ICD) in cancer cells that enables anticancer immunity [54,55,56,57]. ICD-inducing anticancer therapies can achieve this rare pro-immune phenomenon in cancer due to the spatio-temporally defined emission of danger signals (specific ‘eat me’ or ‘find me’ signals and alarmins), as well as pathogen-response signals like cytokines (i.e., type I interferons/IFNs) and chemokines (e.g., CCL2, CXCL1 and CXCL10) [54,58,59,60,61,62]. Although such findings argue for the prioritization of apoptotic ICD for immuno-oncology paradigms, it is clear that ICD can also operate via other forms of RCD such as necroptosis [59,63,64]. Necroptosis is a form of RCD characterized by a genetically and molecularly defined (rather than typically accidental) necrotic program, thereby eliciting necrosis-associated inflammation [52,65]. This change in the molecular machinery underlying ICD biology has important implications for cancer because resistance to RCD, especially apoptosis, is one of the major hallmarks of cancer and has long been a major obstacle for anticancer therapies [66,67]. For instance, while genes coding for pro-apoptotic caspases (such as *CASP8*, *CASP9* or *CASP3*) are under significantly heavy, loci-specific, negative selection or genetic deletion pressures within most human cancers (Figure 1); yet this is not always the case for necrosome-relevant genes (such as *RIPK1*, *RIPK3* or *MLKL*), which experience random or unspecific genetic deletions at the same rate as background genetic aberration frequency in human cancers (Figure 1). This suggests that targeted inactivation of the caspase-based apoptotic machinery during tumour evolution may render cancer cells more susceptible to necroptosis. Hence, putative immunogenic or inflammatory characteristics of necroptosis can be of significant interest in immuno-oncology. However, because of the highly complex and multi-factorial character of cancer, a clear consensus on the immunobiology of necroptosis in cancer cells has been tough to establish.

Here, we will discuss the various aspects of necroptosis immunobiology focusing on immuno-oncology and cancer immunotherapy. While other forms of necrotic RCD such as ferroptosis (an iron-dependent RCD characterized by accumulation of lipid peroxides) and pyroptosis (a RCD dependent on caspases/granzymes-based, context-dependent, proteolytic activation of pore-forming gasdermin proteins, predominantly triggered by pathogenic stimuli) can elicit reactions in a tumor context; yet, in this review we will focus on necroptosis. Herein, we will first discuss the general (disease-independent) concepts underlying the mechanistic and inflammatory characteristics of necroptosis before exploring its specific role in oncology, immuno-oncology and implications for cancer immunotherapy.

## 2. Mechanisms Underlying Necroptosis: A Broad Overview

**Figure 2:**
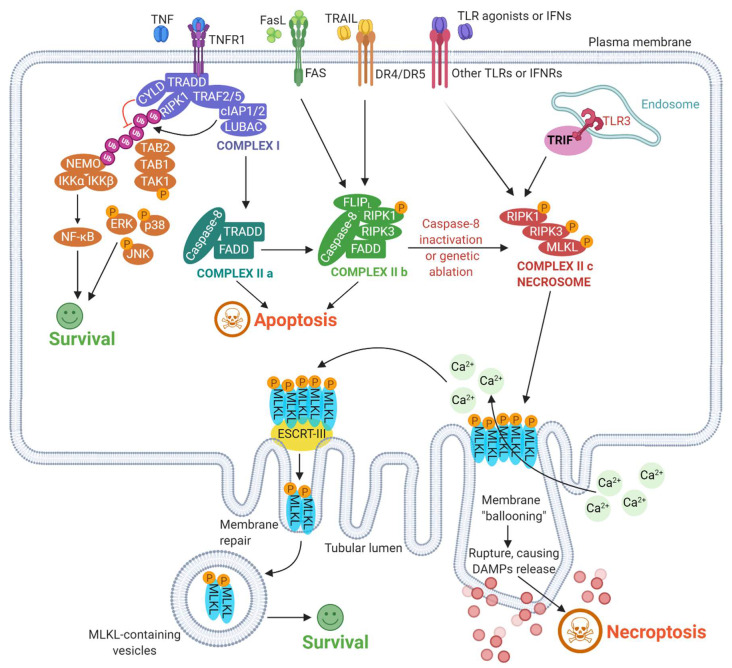
Schematic overview of the mechanisms and cell fate decisions’ crosstalk underlying necroptosis induction. See the text for further details on the pathway. Calcium (Ca^2+^), cellular inhibitor of apoptosis protein 1/2 (cIAP1/2), cylindromatosis (CYLD), death receptor (DR), damage-associated molecular patterns (DAMPs), extracellular signal-regulated kinases (ERK), endosomal sorting complexes required for transport III (ESCRT-III), fas associated via death domain (FADD), FAS ligand (FASL), FLICE-like inhibitory protein (FLIPL), interferon receptor (IFNR), IκB kinase α/β (IKKα/β), c-Jun N-terminal kinase (JNK), linear ubiquitin chain assembly complex (LUBAC), mixed lineage kinase domain like pseudokinase (MLKL), NF-κB essential modulator (NEMO), nuclear factor kappa-light-chain-enhancer of activated B-cells (NF-κB), receptor-interacting serine/threonine-protein kinase 1/3 (RIPK1/3), TAK-1 binding protein 1/2 (TAB1/2), transforming growth factor-β-activated kinase 1/2 (TAK), t-cell receptor (TCR), toll-like receptor (TLR), tumor necrosis factor (TNF), tumor necrosis factor receptor 1 (TNFR1), TNF receptor type1-associated death domain (TRADD), TNF receptor associated factor 2/5 (TRAF2/5), TNF-related apoptosis-inducing ligand (TRAIL), toll/il-1 receptor domain-containing adaptor inducing interferon-β (TRIF), ubiquitinated (Ub).

While necroptosis is a regulated form of molecularly defined necrosis, it resembles accidental necrosis in terms of its final morphology (e.g., organelle swelling, plasma membrane rupture, cell lysis, and leakage of intracellular components) [69,70,71]. Thus, the molecular pathways underlying necroptosis differentiate it from accidental necrosis (and other forms of regulated necrosis) [72,73]. Interestingly, multiple elements of (extrinsic) apoptosis and necroptosis are shared, such as initiating receptor complexes (Figure 2), because the “default setting” for most normal cells is to engage extrinsic apoptosis since it is less likely to be inflammatory [74,75]. However, when extrinsic apoptosis fails to be initiated due to genetic, molecular or pharmacological perturbations (e.g., Figure 1), then the proximal pro-death signals initially meant to trigger apoptosis will now provoke necroptosis [76,77,78,79].

Necroptosis is mostly initiated by the activation of various surface-associated death receptors (DRs) (e.g., tumor necrosis factor receptor 1 (TNFR1), DR4/5, FAS receptor) (Figure 2) [80,81]. Other surface receptors that can initiate necroptosis include pattern-recognition receptors (PRRs), such as Toll-like receptor 3 (TLR3), TLR4, and Z-DNA binding protein 1 (ZBP1) (Figure 2) [82,83]. Downstream of these proximal “initiator” receptors, necroptosis is typically regulated through three major pro-necroptotic molecules, i.e., receptor interacting serine/threonine kinase 1 (RIPK1), RIPK3 and mixed lineage kinase domain-like pseudo kinase (MLKL) (Figure 2) [84,85]. The most well-known inducer of necroptosis is the tumor necrosis factor (TNF) cytokine, which acts via TNFR1 (Figure 2) [86,87,88]. Binding of TNF triggers trimerization of TNFR1 [89,90]. This elicits the formation of “Complex I” in association with the TNF receptor wherein Complex I consists of RIPK1, TNF receptor-associated death domain (TRADD), cellular inhibitor of apoptosis protein 1/2 (cIAP1/2), Cylindromatosis lysine 63 deubiquitinase (CYLD) and TNF receptor-associated factor 2 (TRAF2), among other proteins [91,92,93]. Complex I is an important “checkpoint” for cellular fate (Figure 2). Depending on the context (stress levels, stimuli intensity, signaling amplitude, cell type, disease context, pharmacological agents and physiological situation), Complex I can enable cell survival, apoptosis or necroptosis [82,94,95,96]. For instance, cIAPs can elicit poly-ubiquitination of RIPK1, thereby abolishing any downstream pro-apoptotic signaling and instead facilitating cellular survival (Figure 2). Via this route the activation of the nuclear factor kappa-light-chain-enhancer of activated B cells (NF-κB) and mitogen-activated protein kinase (MAPKs) pathways are initiated [78,82,94,97,98,99,100]. Alternatively, inhibition of cIAPs [e.g., via degradation through second mitochondria-derived activator of caspases (SMAC)] and/or RIPK1 de-ubiquitination via CYLD, can inhibit NF-κB stimulation, causing dissociation of RIPK1/TRADD from the death-receptors complex [101]. This series of events can pave the way for apoptosis induction via a complex called cytosolic death-inducing signaling complex (DISC) (or complex IIa), mainly composed of RIPK1, TRADD, FAS-associated death domain protein (FADD) and procaspase-8, that can lead to caspase-8-driven extrinsic apoptosis (Figure 2) [82]. Similarly, FAS/TRAIL receptors can engage a (variant) complex IIb, wherein activated caspase-8 can undertake cleaving of pro-necroptotic proteins thereby firmly steering the cellular fate in the direction of apoptosis [102,103,104,105]. Thus, in the absence of caspase-8 inhibition, these signaling events largely underlie the balance between survival and apoptosis.

However, when capase-8 is inhibited (e.g., via cellular FLICE-like inhibitory protein long isoform protein (cFLIPL)), disrupted (e.g., via pharmacological or genetic/post-translational ablations) or directly repressed (e.g., due to ultra-high levels of pro-necroptotic RIPK3 and MLKL proteins), the activated RIPK1 in complex IIb is free to recruit RIPK3 thereby facilitating its phosphorylation-driven activation, which in turn recruits and phosphorylates MLKL [106,107,108,109]. This assembly of activated RIPK1, RIPK3 and MLKL enables the formation of the necrosome (or, complex IIc), capable of facilitating necroptosis (Figure 2) [110,111,112,113]. Herein, necroptosis is specifically triggered via necrosome-elicited oligomerization of MLKL [114,115,116]. The activated MLKL then migrates toward the plasma membrane, to create membrane pores (accompanied by “ballooning” of the plasma membrane) that facilitate massive calcium ions (Ca^2+^) influx thereby causing the rupture of the plasma membrane (Figure 2) [117,118,119,120]. Besides the plasma membrane disruption, MLKL may also target the mitochondrial membrane and induce mitochondrial disruption by opening the mitochondrial permeability transition pore (mPTP) [121,122]. This can result in an excessive production of reactive oxygen species (ROS), further amplifying cell death [123]. It has recently emerged that these MLKL activities do not immediately cause plasma membrane rupture. This is because the endosomal sorting complexes required for the transport III (ESCRT-III) machinery strives to control the duration of plasma membrane disruption, allowing the necroptotic cells to sustain their ongoing transcriptional programs for a relatively longer time [124]. This complex process probably exists since, in case MLKL activation is insufficient in sustaining necroptotic signaling or unstable/reversible, than this ESCRT-III machinery can perhaps prolong cellular survival [124].

This clearly shows that most cells are originally programmed to undergo apoptosis while avoiding necroptosis, and only if they fail in this endeavor, do they opt for a necroptotic route of cell demise. Although it must be considered that necroptosis can also be defective in cancer cells (discussed later). Thus, the tendency of human cancers to genetically disrupt caspase-8 (Figure 1) can amply explain their relative susceptibility toward necroptosis. This can also explain why eventually cancer cells can show tendency to disrupt pro-necroptotic machinery (discussed later). It remains to be determined whether caspase-8 deletion is also associated with enhanced (compensatory) deletion of the necroptotic pathway. Irrespective of this, the MLKL-driven plasma membrane rupturing underlies inflammatory characteristics of necroptosis owing to spillage of intracellular contents and immunological signals.

## 3. Necroptosis and Inflammation

Necroptosis, by necrosome-facilitated cellular swelling and collapse of the plasma membrane, culminates into the spillage of its intracellular organelles and biomolecules into the extracellular environment. However these released intracellular biomolecules are not restricted to those constitutively produced, or present before necroptosis, e.g., danger signals such as damage-associated molecular patterns or DAMPs (molecules hidden within normal cells and performing non-immunological functions but liberated upon cell death to perform immunological functions like chemotaxis, phagocytosis and immune cell activation) [125,126,127]. Due to various molecular mechanisms and events discussed above, necroptosis can sustain transcriptional as well as translational activity for a considerable duration even during the necrosome-activity [128,129]. This allows dying cells to produce various (possibly new) necroptosis-specific inflammatory factors and cytokines (e.g., interleukin 6 or IL6) in its penultimate moments [130]. In fact, a recent study proposed that cytokine production may even continue in the necroptotic cells after the loss of plasma membrane integrity at the level of the endoplasmic reticulum (ER), which remains intact for a relatively longer time after cellular disruption [131]. Although it is not clear whether all these cytokines are sufficiently mature or functional, since they skip post-ER maturation steps and thus exist in “native or immature” form [132]. Nevertheless, these insights underscore the heightened inflammatory potential of necroptosis. Moreover, the delay in plasma membrane rupture during necroptosis may enable a relatively “more ordered” removal and degradation of necroptotic cells through the timely recruitment of phagocytic cells [133,134,135]. In the below subsections we discuss the general evidence associated with phagocytic clearance of necroptotic cells and the mechanisms underlying necroptosis-driven inflammation.

### 3.1. Necroptosis: Innate Immune Attraction and Phagocytic Clearance

Rapid and orderly clearance of dying cells (i.e., efferocytosis) is very important for tissue homeostasis [136]. Phagocytes that are specialized to clear them need to prevent an unwanted immune response. This is especially important to limit inflammation in response to intracellular materials that leak out of necrotic and necroptotic cells [137]. Even though necroptotic cells successfully delay their rupturing, this delay is typically not as long as apoptosis. The first phase that precedes efferocytosis mainly involves, dying cells releasing “find me” signals and/or chemokines that attract surrounding phagocytes (i.e., innate immune cells or myeloid cells like macrophages and DCs or granulocytic cells such as neutrophils) [133,134,138,139]. These phagocytes distinguish dying cells from healthy cells via specific engulfment receptors, which recognize “eat me” signals on the surface of dying cells [134,140,141,142]. Upon internalization, degradation and processing, the phagocytes may elicit a specific response by secretion of pro- or anti-inflammatory factors depending on the nature of the ingested cargo [140,143,144].

Necroptosis has not been associated with any specific “find me” signal distinct from other such signals typically secreted or released from various dying/dead cells. For a more detailed description of “find me” signals typically released by dying/dead cancer cells, please refer to other more detailed review articles [59,133,134]. Pending on the cell type, necroptotic cells secrete a variety of chemokines for efficient myeloid/granulocytes recruitment e.g., CCL2, CXCL8/IL8, CXCL1/2, CSF2 [145,146]. Similarly, ubiquitous DAMPs capable of acting as “find me” signals such as extracellular ATP released post-rupture of the plasma membrane, can recruit phagocytes toward cells undergoing necroptosis [63,133,147]. However, it is interesting to note that innate immune cells may differentially react to necrotic or necroptotic cells in terms of chemotaxis, e.g., monocytes and macrophages may show more rapid and sustained attraction towards necrotic cells rather than DCs and neutrophils [60,148]. In fact, the tendency of accidental necrotic cells to preferentially sustain macrophage recruitment may even be evolutionarily conserved across vertebrates [60,149,150]. Nevertheless, the quality as well as the quantity of necroptosis-based innate immune cell recruitment depends on the inflammatory context, type of tissue, fitness of the local circulatory system and overall stress on the target cells. Of note, due to lack of systematic comparative studies, it is currently not clear if there exist strong differences between the recruitment patterns of innate immune cells toward necrotic or necroptotic cells. However, published evidence scattered across different studies suggests a largely overlapping monocytes or macrophages-driven chemotactic response to both cell death modalities [61,136,151]. Nevertheless, more comprehensive studies are required to address this gap-in-knowledge.

Once the innate immune cells have been recruited, they can undertake efferocytosis. Herein, compared to the efferocytosis of apoptotic cells, the clearance of necroptotic cells remains poorly understood. It is proposed that innate immune cells engulf necroptotic cells through macropinocytosis, i.e., endocytosis of small fluid-suspended particles and co-uptake of extracellular fluid, likely generated after the cellular explosion of necrotic or necroptotic cells [152,153]. Yet, several studies have reported specific necrotic cells-linked biomolecules that facilitate direct engulfment via engagement of cognate scavenger receptors on phagocytes, especially DCs, e.g., necrotic cell-associated keratin or F-actin engaging myeloid DEC205 receptor (also called Lymphocyte Antigen 75 or LY75) [154] or DNGR1 receptor (also called C-type Lectin Domain Containing 9A or CLEC9A) [155], respectively. Beyond these necrotic pro-phagocytic entities, necroptotic cells may also expose some canonical “eat me” signals such as phosphatidylserine (PtdSer) [137,147,156,157]. It has been indeed shown that necroptotic cells are engulfed by the macrophages in a PtdSer-dependent manner [158], which underlines that PtdSer is a ubiquitous “eat me” signal mediating engulfment of several RCD modalities. However, the critical evidence supporting exposure of surface calreticulin (CALR) by necroptotic cells is largely absent. Moreover, CD47, a well-known “do not eat me” signal, has been found to modulate phagocytic clearance of necroptotic cells [157,159]. Irrespective of these observations, it is generally accepted that the uptake of necroptotic cells might not be as proficient as that of apoptotic cells [156,157,158,159], although an opposite scenario in which necroptotic cells were efferocytosed more efficiently, has also been proposed [137].

### 3.2. Necroptosis-Driven Modulation of Immune Responses

Necroptosis, as emphasized above, liberates a plethora of DAMPs, while secreting/releasing several inflammatory cytokines and chemokines [65,146]. The DAMPs that are secreted/released upon necroptosis include: (I) “find me” signals (as described above); (II) intracellular chaperones such as heat-shock proteins (HSPs) [160] and S100 protein family members; (III) alarmins or cytokine-like DAMPs [161] and (IV) nucleic acids (nuclear or mitochondrial DNA) [162]. One of the most ubiquitous alarmins released during necroptosis is the high mobility group box 1 (HMGB1) protein [63,163,164]. These DAMPs usually stimulate the initial sensors of infection or damage, i.e., PRRs on myeloid cells, thereby efficiently co-stimulating them and enabling a better interface between these activated myeloid cells and adaptive immune cells [165]. Such a productive myeloid::lymphoid cell interface can activate antigen-specific cytotoxic CD8^+^ T cells that can help in the elimination of antigen-expressing entities or diseased cells [166]. The pro-inflammatory DAMPs are proficiently supported by cytokines or chemokines released from these cells [166], and these factors together coordinate the necroptosis-driven inflammatory response [167].

Production of cytokines during necroptosis is not a “bystander” process, since pro-necroptotic proteins such as RIPK1 (as a scaffold) have been found to directly facilitate production of cytokines such as IL6 [166]. Similarly, comparative transcriptomics analysis showed that TNF-induced necroptosis promotes the expression of pro-inflammatory cytokines in a cell-autonomous fashion, through the direct involvement of pro-inflammatory NF-κB pathways [145]. Interestingly, RIP1/3 can also facilitate inflammasome activation, enabling the stimulation of IL1β and IL18 cytokines [168]. Moreover, there is accumulating evidence that inflammation itself can also regulate necroptosis via cytokines like type I/II IFNs, that can increase the expression of MLKL, which indicates a putative existence of a necroptosis-inflammation auto-fueling loop [169]. However, despite a clear pro-inflammatory phenotype, final immunological outcome of necroptosis may not always be sufficiently immunogenic [158]. This can happen because the simultaneous release of immunosuppressive/tolerogenic as well as homeostatic DAMPs, cytokines or chemokines, can create substantial challenges for a resolved immune response [170]. For instance, concurrent release of such complex and often contradictory modulators of immune responses, at high concentrations, from necroptotic cells has the potential to create a localized “inflammatory storm”-like situation, which is often seen during response to infection [83,171,172]. This can facilitate chronic inflammation thereby eliciting differentiation of immunosuppressive myeloid or lymphoid cells [173,174,175]. This scenario, depending upon the disease context, can be substantially detrimental and impede disease amelioration.

Thus, necroptosis and its immunobiology cannot be interpreted in a “black-and-white” manner and should not be applied in a generalized fashion to all pathophysiological situations. It is extremely important to consider the tissue, stress and disease contexts where necroptosis is positioned as well as the duration or amplitude of inflammation elicited by necroptosis in order to sufficiently decipher its disease aiding or impeding effects. This train of thought is especially applicable to a highly complex disease like cancer that spans nearly all known human organ systems, almost all possible stress scenarios (from tumor-associated physico-chemical stress to a plethora of therapeutic agent classes) and often presents with extremely variable inflammatory profiles (from low-to-high basal inflammation as well as T cells ‘cold’-to-‘hot’ tumors).

## 4. Necroptosis in Oncology

Necrosis within a tumor, especially in the “core” regions typically deprived of proper nutrient and/or oxygen access, is a well-established phenomenon and such tumor necrosis is classically associated with poor patient prognosis [176,177,178]. Accordingly, several peripheral biomarkers of tumor-associated necrosis, such as serum-associated lactate dehydrogenase (LDH) levels, also tend to have a negative prognostic impact in cancer patients [179,180]. A wide array of stressors can elicit regulated necrosis (e.g., necroptosis) within a tumor (based on largely experimental rather than real-time clinical data) e.g., hypoxia, nutrient starvation, acidosis (due to dysregulated vasculature), conventional anticancer therapies administered at high doses and cytokines or extrinsic cell death-inducing ligands-based pro-necroptotic stimuli produced by myeloid or lymphoid cells infiltrating the tumors (e.g., TNF, IFNs, FAS ligand) [20,174,181]. In Table 1, we summarize several different chemical or biological therapeutic agents that have been consistently reported to induce necroptosis in various human cancer cells (in experimental conditions). Interestingly, some widely used conventional anticancer therapies have demonstrated pro-necroptotic activities like platinum-based chemotherapy (cisplatin) and proteasome inhibitors (bortezomib) (Table 1).

However, the connections between necroptosis (or for that matter any necrotic RCD) and tumor-associated histopathological necrosis cannot be linearly assumed since in the human tumor context, it is nearly impossible to discriminate secondary necrosis, accidental necrosis and regulated forms of necrosis. This is in part because antibodies against typical pro-necroptotic molecules (e.g., phosphorylated forms of RIPK1/3 or MLKL) do not work reliably in the settings of clinically relevant immuno-histochemistry [199,200]. All of this makes it nearly impossible to benchmark and monitor necroptosis in routine human tumor tissue samples. To compensate for this severe disparity, over the years, researchers have relied on proxy approaches such as exploiting the genetic expression of pro-necroptotic molecules (especially *RIPK3* or *MLKL* genes) as well as their univariate or multivariate impacts on human cancer patient’s survival or anticancer therapy responsiveness to study their correlations with inflammatory or immunological parameters. Interestingly, such studies have indicated that even though pro-necroptotic genes may not be selectively deleted or mutated (as evident from our analyses; Figure 1), yet their expression can still be suppressed via epigenetic mechanisms. For instance, epigenetic events (mostly DNA methylation-driven) can cause RIPK3 protein suppression in several human cancer cell lines, including breast cancer [201], colorectal cancer (CRC) [202] and acute myeloid leukemia (AML) [203] (in nearly 2/3rd of >60 human cell lines tested). Interestingly, genome-wide analysis identified that such DNA methylation-driven suppression of RIPK3 levels was oncogene-enforced and accordingly facilitated cancer cells’ ability to avoid necroptosis. Remarkably, this loss of RIPK3 expression was gradually (rather than abruptly) achieved during tumor progression, both in patient tumor biopsies as well as xenograft tumor models, thereby suggesting a role for negative selection pressures against RIPK3 [204]. In Table 2, we summarize the three major RIPK3 “silencing” tactics employed by cancer cells according to recent research. DNA hypomethylating agents can restore RIPK3 expression in cancer cells, “rescuing” their necroptotic capacities and enhancing various beneficial effects of chemotherapy, including promotion of anti-tumor immunity [201].

Although these mechanistic observations vouch for an anticancer role for pro-necroptotic molecules, and possibly necroptosis, yet independent biomarker-based studies to decipher the prognostic or predictive impact of pro-necroptotic genes in cancer patients have not painted a consistent picture. For instance, while multiple studies have indeed found that, whole tumor-biopsy level, high expression of *RIPK3* or even sometimes *MLKL* may predict prolonged cancer patient survival (Table 3), yet high expression of *RIPK1* or *MLKL* has also been documented to predict worse prognosis for some cancer patients (Table 3). We have summarized in Table 3, various major clinical studies exploring the prognostic/predictive biomarker impact of these pro-necroptotic molecules in cancer patients. Overall, the relationship of pro-necroptotic molecules’ gene-expression with cancer patient’s prognosis seems to be highly context-dependent. For example, the analysis of *RIPK3* expression in 74 primary CRC tumors treated with first-line 5-fluorouracil (5-FU) chemotherapy revealed that high expression levels of *RIPK3* correlated with a significantly longer overall survival and lower risk of disease progression in CRC patients [208]. Consistent with this, low expression of *RIPK1* was correlated with, higher tumor progression in head and neck squamous cell carcinoma (HNSCC) patients [209] and worse prognosis in human hepatocellular carcinoma (HCC) patients [210]. In contrast to this, higher expression of *RIPK1* was also linked to a worse prognosis and higher metastasis rate in melanoma, potentially due to NF-κB-dependent stimulation of cancer cell proliferation [211]. Finally, a dual prognostic role for *MLKL* gene expression also emerges from the summary presented in Table 3. For instance, while in breast tumors, high *MLKL* expression associated with poor prognosis, yet in CRC low *MLKL* expression predicted shorter overall survival in the patients. Of note, an extensive number of studies using a diversity of murine tumor models (spontaneous as well as transplantable) have also mirrored these clinical dichotomies. Indeed, several studies demonstrate either a pro-tumor or anti-tumor role for pro-necroptotic genes (frequently tested through genetic knock-out or knock-down models) and/or necroptosis (induced via TNF/TRAIL-based ‘cocktails’ or genetic means). Due to a largely clinical focus of this review, we do not discuss these studies in detail except those with clear immunological basis (see below). Yet, we would like to refer the reader to other excellent reviews on this topic [212,213,214,215,216].

In agreement with findings from primary tumors, the role of necroptosis in tumor metastasis (in clinical as well as experimental settings) also seems to be a double-edged sword [217]. For instance, in metastatic lung cancer, loss of *RIPK3* was documented to reduce the number of tumor nodules by 38% [218]. However, in contrast, high *MLKL* expression in cervical SCC has been associated with low histological grade and lymphatic metastasis such that patients with low expression of *MLKL* exhibited poor prognosis [219]. Similarly, various experimental studies have highlighted the ability of tumors to exploit necroptosis for facilitating metastases. For instance, cancer cells can induce necroptosis in endothelial cells and thereby enable metastasis [220]; or, necroptotic pancreatic cells can upregulate CXCL5 and CXCR2 to facilitate their migration and invasive abilities [221].

We believe that the relative inconsistencies in the prognostic/predictive impact of the pro-necroptotic genes may stem from several complicated reasons. Beyond the obvious variations due to cancer-types, organ-of-origin for different tumors and disparate therapeutic or non-therapeutic contexts from which such analyses were derived, also non-necroptotic functions of MLKL or RIPK1/3 (e.g., RIPK1 as a scaffold/survival or RIPK1/3 as pro-apoptotic factors) could contribute to such inconsistencies. Especially for RIPK1/3, myriads of pathophysiological and inflammatory functions completely unrelated to necroptosis have recently emerged [110]. For MLKL such knowledge is scarce, but few publications have indicated a dynamic profile of non-necroptotic functions [119,222]. Besides that, most of the studies highlighted in Table 3 were done on bulk tumor tissue samples, wherein it is not clear from which source (i.e., cancer cells, immune cells, fibroblasts or other stromal cells), or cellular states (e.g., hypoxia, acidosis, proliferation) these gene expression profiles originate. For example, it is known that hypoxia induces anaerobic glycolytic metabolism and reduces RIPK1/RIPK3 expression thereby conferring resistance to necroptosis [223]. This is crucial because RIPK1/3 or MLKL as well as necroptosis can have very different functional impacts depending on their cells-of-origins. For instance, RIPK1 is heavily expressed by tumor-associated macrophages (TAMs) in pancreatic cancer wherein RIPK1 facilitates TAMs-driven immunosuppression (possibly via its scaffolding functions) [224]. In fact, these observations instigated the testing of pharmacological inhibitors of RIPK1 in pancreatic cancer patients (with or without combination with anti-PD1 immunotherapy) with the aim to ameliorate the immunosuppressive tumor milieu [224]. Thus, we believe a detailed tumor tissue analyses via single-cell RNA sequencing (scRNAseq) that can simultaneously capture the cells-of-origin for these pro-necroptotic genes and their stress-related (genetic) contexts, will be instrumental in solving some of these conundrums. However, nevertheless, the prognostic or predictive role of necroptosis in oncology will remain controversial until a specific biomarker or necroptosis-labelling/profiling technology is discovered, that can consistently specifically identify necroptosis within the complex human tumor tissues and discriminate it from other regulated necrotic cell death modalities.

## 5. Necroptosis in Immuno-Oncology

### 5.1. Necroptosis-Driven Modulation of Anticancer Immunity

The tumor immune landscape, unlike the landscape of other immunopathological diseases, cannot be easily integrated within a single paradigm [234,235]. Even at a very broad level, the tumor immune landscapes tend to be distinguished by highly contrasting features like lymphocyte/IFNγ-dominance, lymphocyte-depletion, immunological silence, inflammation, wound-healing enrichment or TGFβ responses [236]. This is further complicated by sharply different molecular (e.g., antigenicity vs. immunogenicity), cellular (e.g., myeloid vs. lymphoid enriched or depleted) and tissue-level (e.g., immune cells enriching or excluding environments) disorganizations. Herein, necroptosis in cancer cells is expected to mediate cancer-relevant immune responses by facilitating interactions between dying cancer cells and immune cells through the release of DAMPs, cytokines and/or chemokines within the tumor microenvironment [139,153,237]; however, such unidirectional immune response seldom exists.

**Figure 3:**
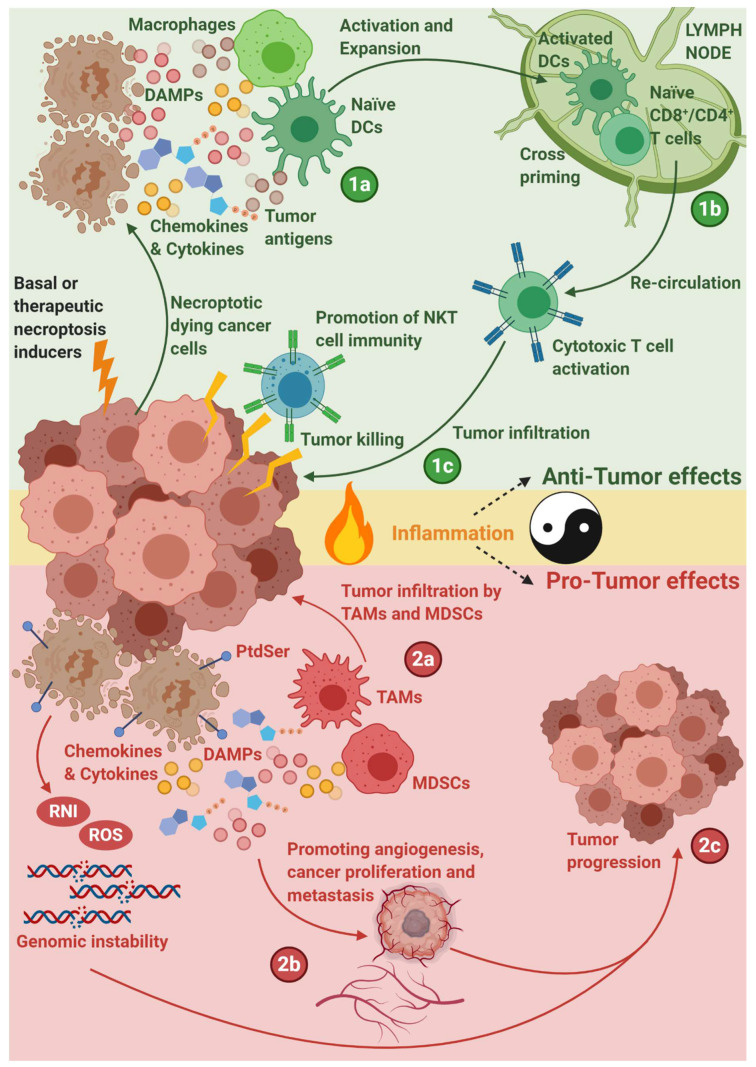
A schematic overview of necroptosis driven pro-tumor or anti-tumor immune responses. Necroptotic cancer cells release, damage-associated molecular patterns (DAMPs), chemokines, cytokines, and/or cancer antigens [and can also surface-expose phosphatidylserine (PtdSer)], which creates an inflammatory immune microenvironment that can either have anti-tumor or pro-tumor effects. In the former scenario, necroptotic cancer cells may attract macrophages and naïve dendritic cells (DCs), that can get activated by necroptosis-derived DAMPs/cytokines (1a). Herein, activated DCs can migrate to the lymph nodes and cross-prime naïve CD8^+^/CD4^+^ T cells for cancer antigens (1b). Upon such interactions, naïve T cells can differentiate into effector cytotoxic T cells and re-circulate out of the lymph nodes to infiltrate the tumor and kill the cancer cells. In parallel, RIPK3 can also induce the expression of cytokines that can activate natural killer T cells (NKT cells) which will also help in killing the cancer cells (1c). However, in the latter scenario, necroptotic cancer cells can also attract myeloid-derived suppressor cells (MDSC), and/or tumor-associated macrophages (TAM) which can cause tumor-associated immune suppression (2a). In parallel, cytokines released by necroptotic cancer cells can also promote angiogenesis, cancer proliferation and metastasis, combined with the release of reactive oxygen species (ROS) and reactive nitrogen intermediates (RNI) thereby facilitating genomic instability (2b), and further contributing toward tumor progression (2c).

Depending on the tumor type, the organ-of-origin for a tumor and immune cell types involved, phagocytic clearance of necroptotic cancer cells may create a spectrum of variable inflammatory responses ranging from facilitation of pro-tumor inflammation to anti-tumor immunity (Figure 3) [238]. Thus, beyond understanding the immunobiology of necroptosis in cancer, it is also necessary to understand what can predict anticancer inflammatory responses elicited by necroptosis in each tumor milieu. Even independently of the cancer context, it is quite clear that the immunological and pathophysiological profile of necroptosis can be contextually variable. Thus, it is not surprising that such variations would also be visible in a complex disease like cancer thereby limiting our ability to unilaterally label necroptosis as a ubiquitously immunogenic form of cell death in cancer. However, an ideal scenario is the one where we can clearly appreciate as well as benchmark the tolerogenic or immunogenic profiles of necroptosis in cancer.

A series of evidence emerging from cancer research involving conventional chemotherapeutics, radiotherapy, anticancer vaccines or oncolytic viruses, shows that necroptosis in cancer can be immunogenic, and even exhibit a clearly discernible ICD-like profile (Figure 3). For example, TNF or chemotherapy (mitoxantrone/oxaliplatin)-driven necroptosis in cancer cells releases several different DAMPs such as ATP and HMGB1, thereby facilitating tumor-regressive anticancer immunity [239]. Moreover, disruption of pro-necroptotic factors in these settings suppresses anti-tumor immunity, thus mechanistically connecting necroptosis with pro-immunogenic effects in cancer [239]. Similarly, ablative radiation therapy can induce necroptosis in non-small-cell lung carcinoma (NSCLC) and mediate HMGB1-driven immune responses [225]. Furthermore, two separate studies have demonstrated that cervical cancer cells, treated with a viral dsRNA analogue (polyI:C), experienced necroptosis accompanied by release of the IL1α cytokine that elicited activation of DCs and DCs-derived IL12-driven immune response [240,241]. In fact, absence of RIPK3 in cancer cells is documented to reduce the type I IFNs-mediated immune responses following treatment with chemotherapy; such that, failure to induce necroptosis (due to lack of RIPK3 or MLKL) can hamper tumor-associated immune infiltration after chemotherapy [239]. In line with this, oncolytic virus (Newcastle disease virus)-elicited necroptosis is also capable of liberating an ICD-like DAMPs profile and propagating potent and long-lasting anti-tumor immunity driven dominantly by antigen-specific CD8^+^ T cells [242]. More refined mechanistic studies wherein necroptosis was encouraged via genetic methods, also recognized that necroptotic cancer cells can trigger CD8^+^ T cells-driven anti-tumor immunity [63,166]. However there was one notable exception among these latter studies that was not reported in therapeutically induced necroptosis studies: contingent on the genetic approach applied for eliciting necroptosis, either DAMPs or NF-κB-driven inflammatory transcriptional program, but not both simultaneously, were observed to mediate the pro-immunological effects of necroptosis [166,214]. Considering the pleiotropic nature of immunologically or therapeutically induced necroptosis, it could be postulated that such differences between DAMPs vs. a NF-κB inflammatory program may get over-represented due to the relatively “cleaner background” of genetically driven necroptosis. Nevertheless, based on ICD studies, both DAMPs and at least some (if not all) NF-κB-based inflammatory factors are crucial for anticancer immunity. Thus, necroptotic modalities that can strike a balance between the two signaling paradigms may have the broadest possible advantage in various tumor contexts.

In several tumor scenarios, where the tumor milieu is depleted of effector immune cells, necroptosis may also fail to elicit strong immunogenic reactions, although in some cases, necroptotic cancer cells can exhibit threshold levels of immunogenicity [49]. While these threshold levels of immunogenicity may not be high enough to autonomously drive tumor regression, yet they may enable other immunotherapeutic paradigms to elicit anticancer immunity. This could be particularly relevant in cancer types that, (I) are enriched in stable/immunodominant antigens [243], (II) have a limited burden of immunosuppressive cytokines [49,63] and/or (III) have augmented mutational burdens due to treatment with genotoxic conventional anticancer therapies [173,244]. Curiously, a recent study reported that, even in absence of a stable or immunodominant cancer antigen, necroptosis-driven anticancer immunity may continue to function (to a certain extent), better than apoptosis [245]. This may highlight the advantages of an “unorganized” antigen uptake (that can be broader) over the more organized form as seen during apoptosis (which could be subject to degradative processes). Moreover, this finding corresponds well with the initial observation that necroptotic cells are internalized (in contrast to apoptotic cells) via macropinocytosis associated with co-uptake of the extracellular content [135,152,246]. These data also underline the importance of cell death-type in defining the amplitude of the immune response. Lessons learned from the recent clinical immuno-oncology scenarios show that translation of necroptosis in human settings is far more likely to encounter immunosuppressive rather than immunosupportive or immunogenic paradigms. Herein, necroptosis does show tendencies to induce tolerogenic immune reactions and/or pro-tumorigenic inflammation (Figure 3), which may also be a “remnant” of its necrotic morphology. For instance, irrespective of the pathway, accidental necrotic cancer cells have disparities in generating long-term antigen-specific CD8^+^ T cell responses [247] and may even release factors that can directly block CD8^+^ T cells’ cross-priming [247]. In line with this, necrotic cancer cells (irrespective of accidental or regulated forms) are broadly known to boost tumor infiltration by immunosuppressive myeloid or lymphoid cells like regulatory T cells (Tregs), anti-inflammatory (M2) macrophages or TAMs and myeloid-derived suppressor cells (MDSCs) [173,181,244]. Similarly, TNF-driven necroptosis in cancer cells can propagate RIPK3-dependent immunosuppression by ablating the release of pro-inflammatory factors, despite proficiently releasing various DAMPs [248]. Necroptosis in cancer can also: exhibit defective recruitment of neutrophils, lymphocytes or monocytes in some contexts [248]; facilitate colitis-driven CRC progression [249]; elicit tolerogenic phagocytic clearance of cellular corpses [147,158]; and show exposure of tolerogenic “eat me” signals such as PtdSer [124,158,250]. These distinctions get further complicated if necroptosis is positioned within a tumor tissue context. For example, IL1α release from necroptotic cancer cells can stimulate the “bystander” stromal cells to proliferate thereby auto-fueling tumor progression via stroma-derived growth factors [20,217,220,251]. Similarly, necroptosis-associated release of reactive nitrogen intermediates (RNI) and/or ROS, at threshold levels, may facilitate pro-tumorigenic processes [20]. Moreover, some chemokines released from necroptotic cells can in fact support pro-cancerous processes, e.g., RIPK3/RIPK1-driven CXCL1 in pancreatic cancer can facilitate tumor progression (via TAMs and MDSCs) by restricting infiltration of highly immunogenic T or B cells [252].

In conclusion, this shows that the contextual nature of necroptosis immunobiology, when merged with the even more complicated tumor immune landscape, can create a highly unpredictable scenario (Figure 3). A potential “two-step” solution to resolve this situation could be, to objectively characterize the immunobiology of a tumor context and then study (therapeutically or immunologically induced) necroptosis in that setting, in order to fully decipher this complex crosstalk. The main experimental challenge in this situation would be to achieve “pure” necroptosis via physiological necroptosis inducers (i.e., cytokines like TNF or IFNs) in an in vivo tumour setting. This can possibly be achieved by creating cancer cells that are simultaneously deficient in major molecules that facilitate other RCD pathways over necroptosis (e.g., caspase-8) and proficient in expressing necroptosis-sensitising proteins (e.g., SMAC overexpression). A combination of advanced genetic engineering techniques (e.g., CRISPR/Cas9) with efficient tumour-targeting cytokine therapies can enable creation of such an ideal experimental setting wherein the immunological significance of necroptotic cancer cells in a tumour can be objectively deciphered. Several such studies in various tumor immune landscapes would ultimately create a clear roadmap to inform decisions on whether to induce or inhibit necroptosis to cause tumor regression. Nevertheless, it is also worth the effort to understand which immunotherapies can be combined with necroptosis to either synergize with it, or overcome its limitations, thereby resulting in long-term anticancer efficacy.

### 5.2. Necroptosis and Combinatorial Cancer Immunotherapy

Immunotherapies targeting immune-checkpoints, as discussed in the introduction section, have revolutionized the field of oncology and led to the creation of an immuno-oncology arm of anticancer treatments in less than a decade since the approval of the first ICB [12,22,253,254,255]. Immune-checkpoints refer to inhibitory pathways of the immune-system that are crucial for maintaining self-tolerance and restraining the overall duration of an immune response [22,256,257]. Since most immune-checkpoints act through extracellular ligand–receptor binding interactions, they can be easily blocked via antibodies acting as ICBs [22]. CTLA4 was the first FDA-approved ICB target, shortly followed by the PD1-PDL1 axis. Since then, ICBs targeting other immune-checkpoints (e.g., TIM3, LAG3, VISTA) have entered several clinical trials [258]. ICBs have indeed markedly improved cancer patients’ outcomes, but several patients still fail to sufficiently respond to these immunotherapies [259]. To overcome this problem, combination of ICBs with existing conventional anticancer therapy are being extensively studied in both clinical as well as pre-clinical studies. The efficacy of ICBs can be substantially boosted when co-administered with cytotoxic therapies that can facilitate effector T cell activity or at the very least, support a pro-inflammatory milieu. Hence, as necroptotic cancer cells can boost pro-inflammatory (myeloid) signals, current endeavors aim to decipher whether necroptosis can successfully synergize with ICBs to create new immunotherapeutics [260]. In mice, combining a biomimetic vaccine composed of an ‘artificial necroptotic cancer cell’ (αHSP70p-CM-CaP) with anti-PD1 antibodies completely regressed tumors and provided long-term anti-tumor immunity [261]. Similarly, inducing spontaneous necroptosis in subcutaneous murine tumors by overexpressing MLKL mRNA synergized efficiently with anti-PD1 immunotherapy to elicit potent anti-tumor immunity [262]. In a very different approach, treatment of mice with ICBs and simultaneous injection of necroptotic fibroblasts (rather than cancer cells themselves) into the tumor, led to improved survival of tumor-bearing mice and induction of long-term anticancer immune memory [260]. These diverse mechanistic studies highlight the putative benefits of combining ICBs with necroptosis in cancer immunotherapy context.

Although the highly artificial nature of necroptosis induction in most of these studies limits their direct translational potential in human settings, yet it would be interesting to test whether tumor necrosis achieved by pro-necroptotic anticancer therapies (Table 1) associates with better responses to ICBs in multi-arm clinical trials. It is worth mentioning here that the serum LDH level, a typical marker of widespread tumor cell death and a negative prognostic biomarker in basal conditions, tends to associate with positive clinical responses to ICBs in melanoma. Thus, it would be interesting to systematically decipher whether these observations may fulfil an unknown benefit of necrosis/necroptosis in ICBs-based treatment settings.

## 6. Concluding Remarks

It is amply clear that necroptosis and its immunobiology cannot be applied to complex pathologies such as cancer in a “black-and-white” or generalized manner. While understanding necroptosis-driven immune responses is crucial, yet it is equally important to understand where, or when, do such immune responses work or fail. Expecting necroptosis to work consistently as an immunogenic pathway in all tumor contexts is probably a faulty strategy. Understanding the differences in necroptosis-associated responses due to tumor tissue (localization or origin), the nature of necroptosis-inducing stress and type of immune cells contacting the necroptotic cancer cells will be the key to unravel its disease-aiding or -impeding effects. Moreover, the current inability to discriminate necroptosis from other forms of regulated necrotic cell death modalities in oncological settings makes it extremely difficult to appreciate the real impact of necroptosis. Thus, there is a strong and urgent need for real-time and reliable biomarkers that can identify necroptosis in human tumor tissue which will help to specifically discriminate it from other forms of regulated cell death modalities. Until such biomarkers are discovered, the immunological and prognostic role of necroptosis in oncology will remain highly controversial. In addition, it has been also shown that necroptosis is often associated with inflammation which, in some cases, could contribute to progression of cancer and promote resistance to anticancer treatments. For example, recently several studies have shown that necroptosis triggers chronic colon inflammation that can facilitate colon cancer development [263,264]. Therefore, knowledge of the precise molecular mechanisms and signaling events which will define the role of necroptosis as cancer inducer and the role of necroptosis as cancer eradicator is highly needed, to allow better development of novel therapeutic approaches in a disease- and tissue- dependent manner. Finally, several studies demonstrating the immunological effects of necroptosis in cancer are still based on in vitro experiments or on murine allograft/xenograft tumor models, thereby increasing the urgency to create human translation studies to systematically understand the therapeutic utility of necroptosis in immuno-oncology.

## Figures and Tables

**Table 1:** Chemical or biological therapeutic agents capable of inducing necroptosis in cancer.

Therapeutic Agent	Cancer Type	Pro-Necroptosis Roles	Refs.
AdipoRon	Human pancreatic cancer cells	Induces necroptosis through p38, MAPK and RIPK1 activation.	[182]
Bromocriptine	Prolactinoma	Cell death induced by bromocriptine, which is a dopamine antagonist, relies on necroptosis	[183]
BV6 + dexa co-treatment	ALL (acute lymphoid leukaemia)	Cell death depends on RIPK3 and MLKL.	[184]
BV6 and Bortezomib	B-cell non-Hodgkin Lymphoma	Induction of necroptosis, even if apoptosis is blocked.	[185]
Ceramide nanoliposomes	Ovarian cancer cell xenograft model	Suppressed metastatic growth through inducing necroptosis	[186]
Cisplatin	Oesophageal cancer	RIPK3 regulates cisplatin sensitivity and could predict chemosensitivity.	[187]
Cisplatin	Lung cancer	Cisplatin induces both apoptosis and necroptotic-like cell death in lung cancer cells.	[188]
Miconazole	Breast cancer cells	Inhibits the proliferation and induces apoptosis and necroptosis.	[189]
Neoalbaconolol	Human nasopharyngeal carcinoma cells	Induces necroptosis by remodeling cellular energy metabolism.	[190]
Oncolytic viruses	Various cancer-types	Mechanism unknown.	[191]
Proteasome inhibitors	Glioblastoma	Proteasome inhibitors and oncolytic HSV induce necroptosis, increase the production of mitochondrial ROS and JNK phosphorylation and significantly enhance NK cell activation.	[192]
Shikonin	Lung cancer, triple negative breast cancer and glioma	Induces necroptosis in cancer cells.	[193,194,195]
Silver nanoparticles	Pancreatic ductal adenocarcinoma	Silver nanoparticles have the potential to overcome barriers involved in chemotherapy failure.	[196]
SMAC mimetic (BV6)	AML (acute myeloid leukaemia)	Sensitizes cell to apoptosis and necroptosis. RIPK1 seems to play the major role in AML.	[197]
SMAC mimetic (LCL161)	Drug resistant breast cancer	Activation of the RIPK1-RIPK3-MLKL necroptosis.	[198]

**Table 2:** RIPK3 epigenetic silencing methods employed by cancer cells.

Silencing Method	Mechanism	Refs.
Epstein Barr Virus (EBV)	EBV infection suppresses RIPK3 expression via hypermethylation of the RIPK3 promotor.	[205,206]
Methylation	RIPK3 can be silenced in cancer cells due to genomic methylation close to its transcriptional start site, thereby inhibiting RIPK3-dependent necroptosis by chemotherapeutics.	[201,207]
Sp1	Zinc-finger transcription factor, named Sp1, regulates the expression of RIPK3 in a direct way. The knockdown of this transcription factor decreases the transcription of RIPK3 and *vice versa*.	[207]

**Table 3:** Biomarker performance of pro-necroptotic genes in oncology.

Expression	Cancer	Prognosis	Refs.
*RIPK3* expression			
High expression	Non-small cell lung cancer	Improved local control and progression-free survival in treatment with hypofractionated radiation therapy (HFRT)	[225]
	Primary CRC (colon rectal cancer)	Longer mean overall survival after treatment with 5-fluorouracil (5-FU)	[208]
Low expression	Breast cancer	Worse prognosis	[201]
	Colon rectal cancer (CRC)	Worse overall survival and disease-free interval.	[202]
*RIPK1* expression			
Low expression	Head and neck cancer (HCC)	Worse prognosis	[209]
	Head and neck cancer (HCC)	Worse prognosis	[210]
High expression	Breast cancer	Promotes metastasis	[226]
	Glioblastoma	Worse prognosis	[227]
*MLKL* expression			
Low expression	Colorectal cancer	Decreased overall survival in treatment with adjuvant chemotherapy.	[228]
	HR-HPV cervical cancer (high risk- human papillomavirus)	Decreased overall survival and disease-free survival	[229]
	Ovarian cancer	Decreased overall survival.	[230]
	Pancreatic adenocarcinoma	Decreased overall survival in patients with resected tumor and decreased RFS and OS in the subset of patients with resected tumors who receive adjuvant chemotherapy.	[231]
High expression	Breast cancer	Worse prognosis.	[232]
	Cervical SCC (squamous cell carcinoma)	Dual: Higher *MLKL* expression in cervical SCC than in normal tissue. Though low expression in cervical SCC indicated poor prognosis.	[219]
	Gastric cancer	Tumor suppressing and a potential prognostic biomarker.	[233]
